# Methamphetamine Enhances HIV-1 Replication in CD4^+^ T-Cells via a Novel IL-1β Auto-Regulatory Loop

**DOI:** 10.3389/fimmu.2020.00136

**Published:** 2020-02-07

**Authors:** Kaycie S. Lawson, Anil Prasad, Jerome E. Groopman

**Affiliations:** Division of Experimental Medicine, Beth Israel Deaconess Medical Center and Harvard Medical School, Boston, MA, United States

**Keywords:** HIV, methamphetamine, inflammation, innate immunity, miRNA

## Abstract

Methamphetamine (Meth) abuse is a worldwide public health problem and contributes to HIV-1 pathobiology and poor adherence to anti-retroviral therapies. Specifically, Meth is posited to alter molecular mechanisms to provide a more conducive environment for HIV-1 replication and spread. Enhanced expression of inflammatory cytokines, such as Interleukin-1β (IL-1β), has been shown to be important for HIV-1 pathobiology. In addition, microRNAs (miRNAs) play integral roles in fine-tuning the innate immune response. Notably, the effects of Meth abuse on miRNA expression are largely unknown. We studied the effects of Meth on IL-1β and miR-146a, a well-characterized member of the innate immune signaling network. We found that Meth induces miR-146a and triggers an IL-1β auto-regulatory loop to modulate innate immune signaling in CD4^+^ T-cells. We also found that Meth enhances HIV-1 replication via IL-1 signaling. Our results indicate that Meth activates an IL-1β feedback loop to alter innate immune pathways and favor HIV-1 replication. These observations offer a framework for designing targeted therapies in HIV-infected, Meth using hosts.

## Introduction

Methamphetamine (Meth) is an illicit drug abused worldwide, posing major public health challenges (1, 2). Meth use increases the spread and replication of Human Immunodeficiency Virus (HIV-1) by fostering risky sexual behaviors, and by facilitating viral infection and rapid progression to AIDS (3–7). Furthermore, Meth abuse is associated with poor adherence to anti-retroviral therapies (8, 9). A clearer understanding of the mechanisms that mediate the molecular effects of Meth on immune defenses should enhance the design of therapies targeted to patients who abuse the drug.

Distinct from HIV-1 infection, Meth has also been shown to alter the expression of inflammatory cytokines in several murine tissues, in the serum of self-administering rats, and in the plasma of human subjects in early recovery from addiction (10–12). Specifically, Meth increased Interleukin-1β (IL-1β) mRNA and protein expression in dendritic cells, and in the rat hypothalamus (13, 14). Chronic increased expression of IL-1β can result in deleterious over-stimulation of the innate immune response, and tissue damage (15). Fine-tuning the innate immune response is crucial for effective immunity. Type I Interferons (IFNs) have been shown to participate in a counter-regulatory antagonistic relationship with IL-1β to maintain the balance necessary for innate immunity (15). Interestingly, Meth suppressed expression of IFNα, a type I IFN, in macrophages (4). Notably, increased IL-1β expression and down regulation of IFN stimulated genes, including TRAF6, are associated with enhanced HIV-1 infection and replication (4, 16, 17). MicroRNAs (miRNAs) participate in transcriptional and translational regulation, cellular homeostasis and feedback regulation, and can serve as indicators of disease states (18–21). For example, miRNA signatures have been established as markers for different cancers, and can act either as oncogenes or tumor suppressors (22). miRNAs contain a highly conserved sequence at their 5′ end, known as a seed region, which binds a target sequence to cause translational repression and mRNA decay (23). Targeted transcripts are highly sensitive to changes in miRNA expression (23–25). In particular, miR-146a has garnered attention recently for its roles in immune regulation (19, 26–28). miR-146a can target TNF Receptor-Associated Factor 6 (TRAF6) and Interleukin-1 Receptor-Associated Kinase 1 (IRAK1) to suppress innate immune responses via negative feedback regulation (19). TRAF6 and IRAK1 serve as fundamental molecules for effective signal transduction resulting from innate immune stimuli (29). Several studies indicate a role for IL-1β in the induction of miR-146a (30, 31). Furthermore, miR-146a plays an important role in T-cell homeostasis, and overexpression of miR-146a in mice results in an autoimmune-like T-cell profile in the periphery (32). These functions of miR-146a merit its exploration it as a target of Meth in HIV-1 pathobiology.

To date, very little is known about the effects of Meth on miRNA expression outside of the central nervous system (CNS). We found that in CD4^+^ T-cells, miR-146a is up-regulated by Meth exposure in an IL-1β dependent manner. Upon Meth treatment, there was altered expression of miR-146a targets, specifically TRAF6. Furthermore, we observed that Meth activated an IL-1β positive auto-regulatory loop, which resulted in enhanced HIV-1 replication. To our knowledge, this is the first report that Meth induces an IL-1β feedback loop linked to increased HIV-1 replication and dysregulation of key innate immune pathways.

## Materials and Methods

### Cell Culture, HIV-1

CD4^+^ T-cells were isolated from healthy human donor buffy coats by ficoll-paque density gradient followed by negative selection using a CD4^+^ isolation kit (Stem Cell Technologies, Vancouver Canada). Isolated CD4^+^ cells were cultured in RPMI medium containing 10% fetal bovine serum (FBS), and 1% penicillin-streptomycin, referred to hereafter as complete RPMI. For stimulation, complete RPMI was supplemented with phytohemagglutinin (PHA-L) and interleukin-2 (IL-2). Following 3 days of stimulation, the media was changed to complete RPMI supplemented with only IL-2 to proliferate CD4^+^ T-cells.

HIV-1 BaL was obtained from the NIH AIDS Research and Reference Reagent Program, National Institute of Allergy and Infectious Diseases, NIH. The HIV-1 strain BaL was used for infection of CD4^+^ T-cells consistent with guidelines from the NIH AIDS Reagent Program, and previous studies (33, 34).

### Reagents, Treatment of Cells

For methamphetamine (Meth) treatment, cells were plated at an initial density of 2 × 10^6^cells/mL. Meth was administered once per day at a final concentration of 100 μM, consistent with observed concentrations of Meth in samples from Meth abusers (35). For cultures with HIV-1, cells were pretreated with Meth for 24 h, followed by infection with HIV-1. Cells treated with IL-1RA were given 200 or 400 ng/mL IL-1RA as described (Shenandoah Biotechnology Inc., Warwick, PA), followed by treatment with 100 μM Meth 2 h later if applicable. Cells treated with both HIV-1 and IL-1RA were pretreated for 24 h with IL-1RA before becoming infected with HIV-1. Cells treated with IFNα2 were administered 100U/mL IFNα2 (PBL Assay Science, Piscataway, NJ) for 2 h prior to treatment with 100 μM Meth. Cells treated with both HIV-1 and IFNα2 were pretreated for 24 h with IFNα2 before becoming infected with HIV-1.

### Western Blotting (WB)

Primary IRAK1 and TRAF6 antibodies were obtained from Cell Signaling Technology (Danvers, MA). GAPDH was used as a loading control (Santa Cruz Biotechnology, Dallas, TX). Western Blotting was performed as described previously (36). Briefly, cells were collected and lysed, and protein was separated using NuPAGE pre-cast gels (Life Technologies Corp., Carlsbad, CA). Gels were transferred via semi-dry electrotransfer to 0.45 μm nitrocellulose membranes (Bio-Rad Laboratories, Hercules, CA), and probed with designated primary antibodies. Blots were then probed with LI-COR IRDye secondary antibodies and imaged using LI-COR Odyssey CLX according to the manufacturer's instructions (LI-COR, Lincoln, Nebraska). Analysis and relative quantification of gel bands was carried out using ImageJ software (NIH, Bethesda, MD) (37).

### Quantitative RT-PCR (Real Time-Polymerase Chain Reaction)

RNA was isolated from CD4^+^ T-cells using TRIzol™ reagent according to the manufacturer's instructions (Thermo Fisher Scientific, Waltham, MA). DNase treatment was performed using TURBO DNA-free kit (Ambion RNA, Carlsbad, CA). One microgram of RNA was used to prepare cDNA using iScript cDNA synthesis kit (Bio-Rad, Hercules, CA). For miR-146a cDNA, iScript Select cDNA synthesis kit was used for specific amplification using a stem-loop primer method (Bio-Rad, Hercules, CA) (38). RT-qPCR was performed in triplicate for each sample with SYBR green based PowerUp™ SYBR™ Green Master Mix (Thermo Fisher Scientific, Waltham, MA) using 100 ng cDNA. All data were normalized to internal Tata-Box Binding Protein (TBP) control gene, and fold change was calculated using the 2^−ΔΔCt^ method.

Primer Sequences:

**miR-146a** RT-SL (**NR_029701.1**): GTCGTATCCAGTGCAGGGTCCGAGGTATTCGCACTGGATACGACAACCCA
**IL-1β** F (**NM_000576.2**): AGCTGATGGCCCTAAACAGATG
**IL-1β** R (**NM_000576.2**): TTGTCCATGGCCACAACAAC
**IRAK1** F (**NM_001569.3**): AGAAAAGTTGGGAGCATGGC
**IRAK1** R (**NM_001569.3**): TTTTGGACACGCAAGAGGAC
**TRAF6** F (**NM_145803.2**): ACGGAGCGCATAAAACAAGC
**TRAF6** R (**NM_145803.2**): TCAGCCCAGCAATTCAGTTG.

### ELISA

IL-1β ELISA assay was performed according to the manufacturer's protocol using cell culture supernatants (Chondrex, Inc., Redmond WA). Culture supernatants from cells incubated with HIV-1 alone or HIV-1 and Meth/IFNα/IL-1RA were harvested on days 0, 1, 3, and 7. P24 ELISA was performed using the Zeptometrix ELISA kit according to the manufacturer's protocol (Zeptometrix Corporation, Buffalo NY). Supernatants were stored at −80°C.

### Transfection

Transfection of primary CD4^+^ T-cells was carried out using “Nucleofector Kit for T-cells” according to the manufacturer's protocol (Lonza Group, Basel Switzerland). miR-146a-5p mimic and mimic “Negative control #1” (Dharmacon, Lafayette CO) were used for transfection at a concentration of 20 nM. miR-146a-5p stem-loop miRNA inhibitor and stem-loop miRNA inhibitor “Negative control #1” (Dharmacon, Lafayette CO) were used for transfection at a concentration of 40 nM. Transfected cells were harvested at 48 h post transfection.

### Flow Cytometry

Cells untreated or treated with Nigericin, Meth, or IFNα+Meth were subjected to the FAM-FLICA Caspase 1 Assay Kit according to the manufacturer's instructions (Immunochemistry Technologies, Bloomington, MN). Nigericin was used as a positive control at a final concentration of 10 μM (Immunochemistry Technologies, Bloomington, MN). Cells were analyzed using a CytoFlex flow cytometer (Beckman Coulter, Indianapolis, IN).

### Statistics

All experiments were conducted at least in triplicate, using at least three different donors, and the results between experimental groups were analyzed by ANOVA. A *p* < 0.05 was considered to be minimally significant.

### Study Approval

Healthy human donor buffy coats were obtained from the Blood Transfusion Service, Massachusetts General Hospital, Boston, MA, in compliance with the Beth Israel Deaconess Medical Center Committee on Clinical Investigations (CCI) protocol #2008-P-000418/5. Buffy coats were provided at this institution for research purposes without identifiers; therefore, no informed consent was needed. This study was approved by Beth Israel Deaconess Medical Center's CCI, Institutional Review Board, and Privacy Board appointed to review research involving human subjects. The experimental procedures were carried out in strict accordance with approved guidelines.

## Results

### Meth Enhances IL-1β Expression and Caspase-1 Activation in CD4^+^ T-Cells

Meth has been shown to alter inflammatory cytokine expression in several murine and human models, both in the periphery and the CNS (10–12, 39). In particular, Meth has been linked to enhanced IL-1β expression in dendritic cells and in the rat hypothalamus (13, 14). Thus, we first sought to study the effects of Meth treatment on IL-1β expression in CD4^+^ T-cells.

**Figure 1:**
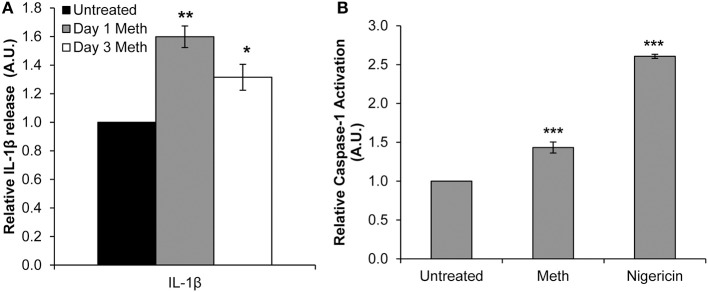
Meth enhances IL-1β expression and Caspase-1 activation in CD4^+^ T-cells. CD4^+^ T-cells were treated daily with or without Meth. **(A)** Expression of IL-1β was determined from cell culture supernatants by ELISA analysis. Relative expression was calculated by normalizing Meth treated samples to untreated control cells. Data represent the mean ± SD of 3 independent experiments, and *p*-values were calculated relative to untreated controls (*p < 0.05, **p < 0.01). **(B)** CD4^+^ T-cells were untreated, treated with Meth, or treated with Nigericin. Caspase-1 Activation was measured using fluorescent labeling with FAM-FLICA, and analyzed by Flow Cytometry. Data represent the mean ± SD of 3 independent experiments, and *p*-values were calculated relative to untreated controls (***p < 0.001).

Healthy donor CD4^+^ T-cells were treated daily with 100 μM Meth, and culture supernatants were harvested on days 1 and 3. We observed significantly increased release of IL-1β on days 1 and 3 of Meth treatment (Figure 1A). These results suggested that IL-1β may be a key cytokine released during Meth exposure.

Two steps are required for IL-1β to become its mature, released form. First, the IL-1β gene is translated to a precursor protein, known as pro-IL-1β (40). Pro-IL-1β undergoes post-translational processing by the NLRP3 Inflammasome and Caspase-1 to yield its mature form (40, 41). Interestingly, Mahajan et al. found that Meth increased expression of IL-1β in dendritic cells, and in microglial cells Meth has been shown to induce activation of the NLRP3 Inflammasome (13, 42). To assess induction of IL-1β processing in Meth treated CD4^+^ T-cells, we analyzed Caspase-1 activation relative to untreated cells 24 h after Meth treatment. Nigericin, a potent microbial toxin known to induce activation of Caspase-1 and the NLRP3 Inflammasome, was used as a positive control. We found that Meth treatment significantly increased the activation of Caspase-1 relative to untreated controls, concordant with increased IL-1β expression (Figure 1B).

### Meth Increases miR-146a Expression and Down-Regulates TRAF6

IL-1β signaling can participate in a positive auto-regulatory loop, resulting in increased transcription of its gene (43). In addition, it has been reported that IL-1β can induce NFκB-dependent miR-146a expression to interfere with innate immune functions (31). Non-coding RNAs play important roles in regulating cellular activities and stress responses. Furthermore, Meth is known to induce activation and nuclear translocation of NFκB (44). Thus, we used RT-qPCR to identify Meth-related changes in miR-146a and IL-1β mRNA in primary CD4^+^ T lymphocytes.

**Figure 2:**
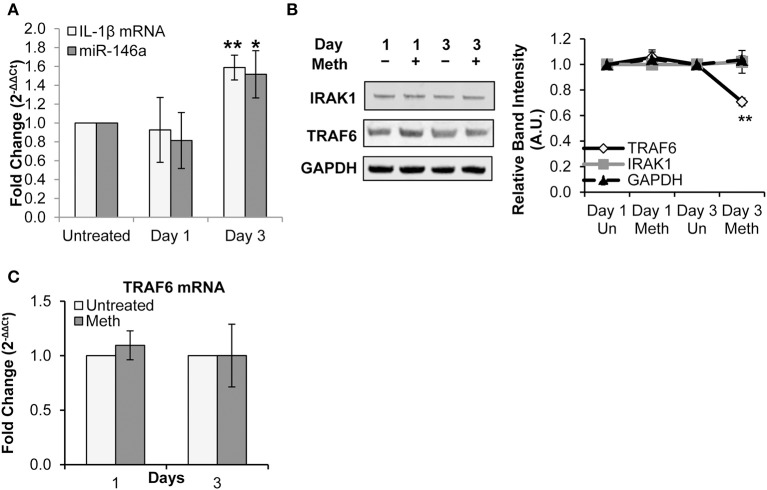
Meth increases miR-146a expression and downregulates of TRAF6. CD4^+^ T-cells were treated daily with or without Meth. **(A)** Expression of miR-146a and IL-1β mRNA was determined by RT-qPCR. Fold change was calculated by normalizing the Meth treated cells to untreated cells. Data represent the mean ± SD of 3 independent experiments, and *p*-values were calculated relative to untreated controls (*p ≤ 0.05, **p ≤ 0.01). **(B)** Cells treated or untreated with Meth were lysed and protein extracts were analyzed for expression of TRAF6 and IRAK1 by Western Blotting. GAPDH was used as a loading control. Relative band intensity was calculated using ImageJ software. **(C)** Expression of TRAF6 mRNA was determined by RT-qPCR. Fold change was calculated by normalizing the Meth treated cells to untreated cells. Data represent the mean ± SD of 3 independent experiments.

Healthy donor CD4^+^ T-cells were treated daily with 100 μM Meth, and miR-146a expression was assessed. We observed that Meth significantly up-regulated miR-146a on day 3 of treatment (Figure 2A). Likewise, we assessed IL-1β mRNA levels in untreated and Meth treated cells. Unlike extracellular IL-1β, which increased after 1 day of Meth treatment, IL-1β mRNA showed increased expression only on day 3 (Figure 2A). Notably, IL-1β release and mRNA expression are controlled by distinct mechanisms (45). In addition, CD4^+^ T-cells constitutively express pro-IL-1β in their cytoplasm (46). As such, our results indicate that Meth first enhances release of mature IL-1β, followed by increased IL-1β gene transcription and miR-146a expression.

We next explored the effects of Meth on TRAF6 and IRAK1, known targets of miR-146a linked to IL-1β and innate immune signaling, and implicated in HIV-1 pathobiology (31, 47). Specifically, IRAK1 plays a key role in mediating IL-1β-induced NFκB activation, and decreased expression of TRAF6 has been implicated in increased HIV-1 replication (17, 47, 48).

By Western Blot analysis, we observed decreased TRAF6 protein expression on day 3 of Meth exposure, but no significant change in IRAK1 protein expression (Figure 2B). Through RT-qPCR, we found no significant change in TRAF6 mRNA expression (Figure 2C).

Collectively, our results— specifically decreased levels of TRAF6 protein and unchanged levels of TRAF6 mRNA— are consistent with fine tuning by miRNA, whereby translational repression precedes mRNA decay (49). Notably, decreased TRAF6 expression observed on day 3 correlated with increased miR-146a levels. These findings suggest a role for miR-146a in regulation of innate immune signaling pathways through repression of TRAF6 during Meth treatment.

We hypothesized that IL-1β signaling is responsible for the increased IL-1β mRNA levels, as well as increased miR-146a expression observed on day 3 of Meth treatment; we further hypothesized that IL-1β-dependent induction of miR-146a would target key innate immune pathways via TRAF6 inhibition.

### Overexpression and Inhibition of miR-146a Confirms Immune Signaling Molecule Targets in CD4^+^ T-Cells

**Figure 3:**
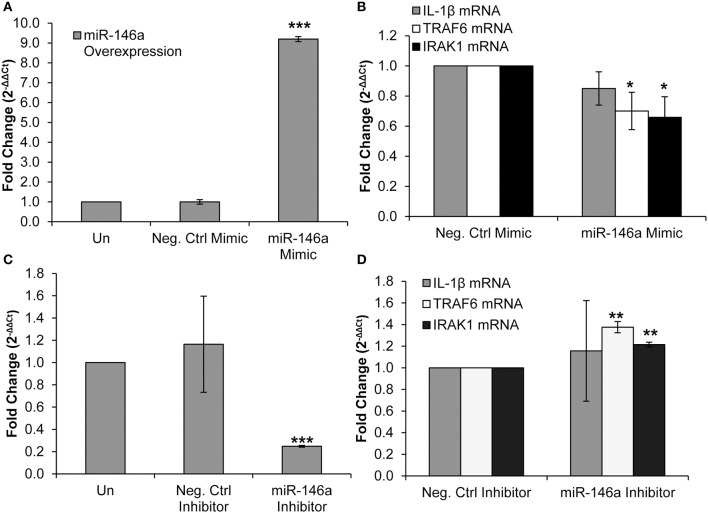
Overexpression and inhibition of miR-146a confirms immune signaling molecule targets in CD4^+^ T-cells. CD4^+^ T-cells were un-transfected, transfected with a negative control mimic or miR-146a mimic. **(A)** Successful miR-146a overexpression was confirmed by RT-qPCR. Fold change was calculated by normalizing the transfected cells to non-transfected cells. Data represent the mean ± SD of 3 independent experiments, and *p*-values were calculated relative to non-transfected controls (***p < 0.001). **(B)** Expression of IL-1β, TRAF6, and IRAK1 mRNA levels during miR-146a overexpression was determined by RT-qPCR. Fold change was calculated by normalizing miR-146a mimic-transfected cells to negative control-transfected cells. Data represent the mean ± SD of 3 independent experiments, and *p*-values were calculated relative to negative controls (*p < 0.05). **(C)** CD4^+^ T-cells were un-transfected, transfected with a negative control inhibitor or a miR-146a inhibitor. Successful miR-146a inhibition was confirmed by using RT-qPCR. Fold change was calculated by normalizing the transfected cells to non-transfected cells. Data represent the mean ± SD of 3 independent experiments, and *p*-values were calculated relative to non-transfected controls (***p < 0.001). **(D)** Expression of IL-1β, TRAF6, and IRAK1 mRNA levels during miR-146a inhibition was determined by RT-qPCR. Fold change was calculated by normalizing miR-146a inhibitor-transfected cells to negative control-transfected cells. Data represent the mean ± SD of 3 independent experiments, and *p*-values were calculated relative to negative controls (**p < 0.01).

TRAF6 and IRAK1 are known direct targets of miR-146a in several cell types, including murine macrophages and liver cells, primary human monocytes, and THP-1 monocytic cells (19, 27, 50, 51). We employed a miR-146a mimic to confirm that TRAF6 and IRAK1 are targets of the miRNA in primary CD4^+^ T-cells. The miR-146a mimic was transfected into CD4^+^ T-cells, resulting in an expected significant increase in miR-146a expression (Figure 3A). We observed no change in IL-1β mRNA expression (Figure 3B). RT-qPCR analysis showed that TRAF6 and IRAK1 mRNA levels were significantly decreased, indicating that miR-146a targeted these molecules and initiated mRNA degradation pathways (Figure 3B).

We then used a miR-146a inhibitor and analyzed changes in TRAF6, IRAK1, and IL-1β mRNA expression. Transfection of the inhibitor significantly reduced miR-146a expression (Figure 3C). IL-1β mRNA expression was unchanged, but TRAF6 and IRAK1 mRNA levels were significantly increased (Figure 3D). Taken together, these data confirm IRAK1 and TRAF6 as targets of miR-146a in CD4^+^ T-cells.

### Meth Increases miR-146a and IL-1β mRNA Expression via IL-1 Signaling

IL-1β has been shown to up-regulate miR-146a expression in THP-1 monocytes by activating its NFκB-dependent transcription (31). Meth treatment of CD4^+^ T-cells increased extracellular IL-1β levels followed by enhanced miR-146a and IL-1β mRNA expression, and decreased TRAF6 protein expression. These results suggested that Meth may modulate the innate immune response via IL-1β signaling to enhance miR-146a and IL-1β mRNA and decrease TRAF6. To address this hypothesis, we blocked IL-1 signaling by employing an IL-1 Receptor Antagonist (IL-1RA).

**Figure 4:**
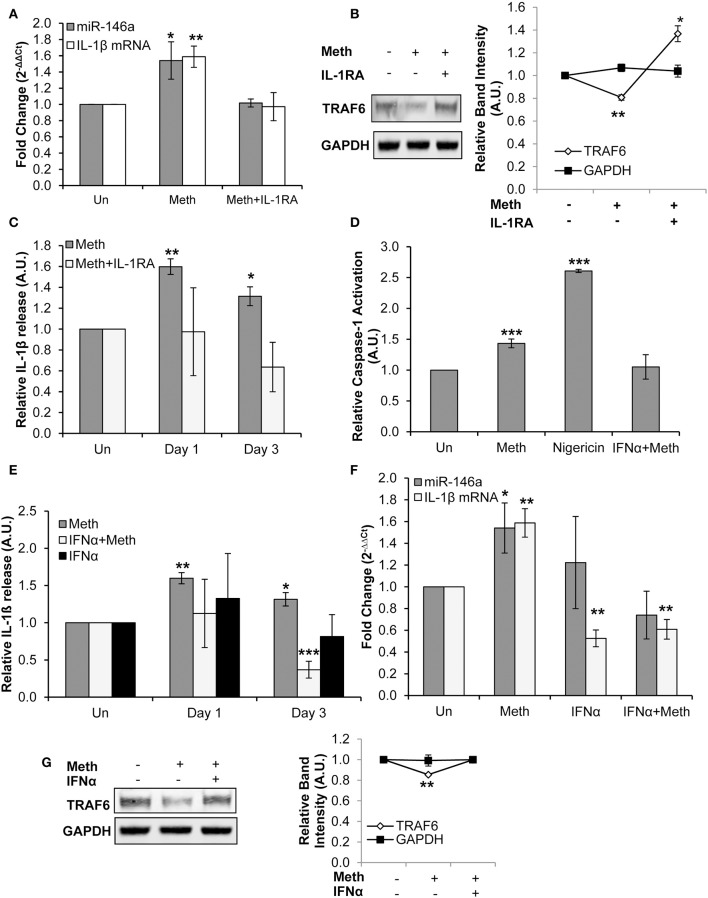
Meth increases miR-146a and IL-1β mRNA expression via IL-1 signaling. **(A)** CD4^+^ T-cells treated with or without Meth for 3 days and IL-1RA were analyzed for miR-146a and IL-1β mRNA expression by RT-qPCR. Fold change was calculated by normalizing Meth treated and Meth+IL-1RA treated cells to untreated controls. Data represent the mean ± SD of 3 independent experiments, and *p*-values were calculated relative to untreated controls (*p < 0.05, **p < 0.01). **(B)** Protein extracts from cells treated for 3 days with or without Meth and IL-1RA were analyzed for TRAF6 by Western Blotting. GAPDH was used as a loading control. Relative band intensity was calculated using ImageJ software, and *p*-values were calculated relative to untreated controls (*p < 0.05, **p < 0.01). **(C)** Culture supernatants were harvested after 3 days of treatment and analyzed for IL-1β by ELISA. Relative expression was calculated by normalizing Meth and Meth+IL-1RA treated samples to untreated controls. Data represent the mean ± SD of 3 independent experiments, and *p*-values were calculated relativeto untreated controls (*p ≤ 0.05, **p ≤ 0.01). **(D)** CD4^+^ T-cells were untreated, treated with Meth, treated with Nigericin, or treated with IFNα and Meth for 24 h. Caspase-1 Activation was measured using fluorescent labeling with FAM-FLICA, and analyzed by Flow Cytometry. Data represent the mean ± SD of 3 independent experiments, and *p* values were calculated relative to untreated controls (***p < 0.001). **(E)** CD4^+^ T-cells were untreated, treated with Meth, or treated with IFNα and Meth, daily for 3 days. Culture supernatants were analyzed for IL-1β expression by ELISA. Relative expression was calculated by normalizing Meth treated samples to untreated controls. Data represent the mean ± SD of 3 independent experiments, and *p*-values were calculated relative to untreated controls (*p < 0.05, **p < 0.01, ***p < 0.001). **(F)** Cells were untreated, treated with Meth, or treated with IFNα and Meth, daily for 3 days. miR-146a and IL-1β mRNA expression were determined by RT-qPCR. Fold change was calculated by normalizing Meth treated and Meth+IFNα treated cells to untreated controls. Data represent the mean ± SD of 3 independent experiments, and *p*-values were calculated relative to untreated controls (*p < 0.05, **p < 0.01). **(G)** Cells were untreated, treated with Meth, or treated with IFNα and Meth, daily for 3 days. Protein extracts were analyzed for TRAF6 by Western Blot. GAPDH was used as a loading control. Relative band intensity was calculated using ImageJ software, and *p*-values were calculated relative to untreated controls (**p < 0.01).

IL-1RA abrogated both Meth induced miR-146a overexpression and increased IL-1β mRNA levels (Figure 4A). Furthermore, TRAF6 protein expression levels, which decreased in the presence of Meth alone, were increased in Meth+IL-1RA treated samples relative to controls (Figure 4B). By ELISA analysis, we observed unchanged extracellular concentrations of IL-1β in samples treated with Meth+IL-1RA, whereas Meth alone resulted in significantly increased extracellular IL-1β levels (Figure 4C). These results support the hypothesis that IL-1 signaling mediates Meth induced miR-146a to target TRAF6. Furthermore, abrogated levels of IL-1β mRNA in cells treated with IL-1RA support the role of IL-1β signaling in a positive auto-regulatory loop.

IFNα, a member of the Type I IFN family, has been shown to negatively regulate IL-1β expression, resulting in a dynamic antagonistic relationship between these cytokines (15). This occurs because Type I IFNs can inhibit Caspase-1 and Inflammasome activation (52). We observed that Meth enhanced Caspase-1 activation in CD4^+^ T-cells, and thus explored the effects of exogenous IFNα on the activation of Caspase-1 in CD4^+^ T-cells.

CD4^+^ T-cells were untreated, treated with Nigericin, treated with Meth, or treated with Meth and IFNα concomitantly (Meth+IFNα) for 24 h. The cells treated with Meth alone showed increased Caspase-1 activation; IFNα abrogated this effect, consistent with the antagonistic relationship between IFNα and Caspase-1 (Figure 4D). Furthermore, by ELISA analysis, we examined IL-1β release under each condition. In the presence of IFNα, there was no change in extracellular IL-1β levels on day 1, but there was significantly decreased IL-1β release on day 3 (Figure 4E). These results show that IFNα inhibits release of IL-1β in Meth treated CD4^+^ T-cells by inhibiting Caspase-1 activation.

After establishing the inhibitory effects of exogenously added IFNα on Caspase-1 activation and IL-1β release, we evaluated its effects on Meth mediated IL-1β mRNA and miR-146a overexpression. While Meth alone significantly increased miR-146a and IL-1β mRNA expression, when cells were treated with IFNα+Meth, miR-146a expression was unchanged and IL-1β mRNA levels were significantly decreased (Figure 4F). Further, we analyzed the expression of TRAF6 protein. Consistent with our earlier results, we observed decreased expression of TRAF6 in Meth treated cells, but IFNα abrogated this decrease (Figure 4G).

Exogenous IFNα counteracted Meth induced Caspase-1 activation and overexpression of IL-1β, in agreement with previous reports that IFNα antagonizes IL-1β (15, 53). The abrogation of Meth mediated miR-146a overexpression by IFNα further supports the role of IL-1β in Meth mediated miR-146a overexpression.

### Meth Enhances HIV-1 Replication via IL-1 Signaling in CD4^+^ T-Cells

Since Meth was found to induce IL-1β and miR-146a expression, and these play important roles in immune regulation, we explored their effects on HIV-1 infection of CD4^+^ T-cells.

**Figure 5:**
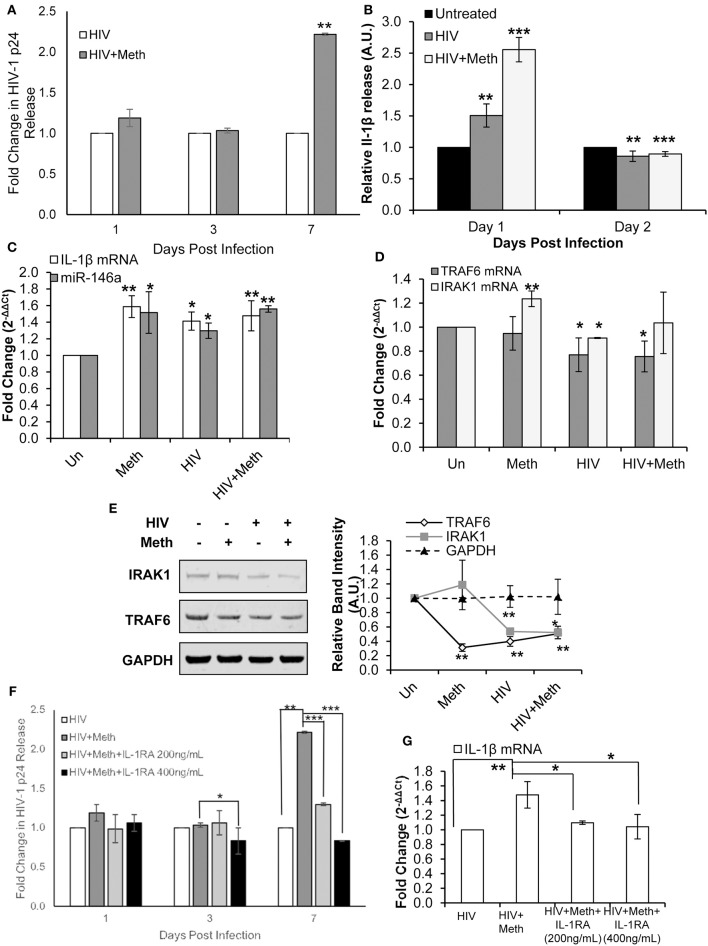
Methamphetamine enhances HIV-1 replication via IL-1 signaling in CD4^+^ T-cells. CD4^+^ T-cells were uninfected, infected with HIV-1, treated with Meth alone, or treated with Meth and infected with HIV-1 concomitantly. Cells were harvested at 2 days post infection and supernatants were harvested at the time points indicated on graphs. **(A)** Culture supernatants were analyzed for HIV-1 replication by p24 ELISA, and *p*-values were calculated relative to untreated controls (**p < 0.01). **(B)** IL-1β ELISA was used to determine the concentration of IL-1β from culture supernatants harvested on days 1 and 2 P.I. Relative expression was calculated by normalizing HIV and HIV+Meth samples to untreated controls. Data represent the mean ± SD of 3 independent experiments, and *p*-values were calculated relative to untreated controls (**p < 0.01, ***p < 0.001). **(C)** miR-146a and IL-1β mRNA expression was determined by RT-qPCR. Fold change was calculated by normalizing Meth, HIV, or HIV+Meth samples to untreated controls. Data represent the mean ± SD of 3 independent experiments, and *p*-values were calculated relative to untreated controls (*p < 0.05, **p < 0.01). **(D)** RT-qPCR was performed to assess changes in TRAF6 and IRAK1 mRNA expression on day 2 P.I. Fold change was calculated by normalizing Meth, HIV or HIV+Meth samples to untreated control cells. Data represent the mean ± SD of 3 independent experiments, and *p*-values were calculated relative to untreated controls (*p < 0.05, **p < 0.01). **(E)** Cells were harvested and lysed on day 2 P.I. Protein extracts were analyzed for IRAK1 and TRAF6 by Western Blot. GAPDH was used as a loading control. Relative band intensity was calculated using ImageJ software, and *p*-values were calculated relative to untreated controls (*p < 0.05, **p < 0.01). **(F)** CD4^+^ T-cells were infected with HIV-1 alone, infected with HIV-1 and treated with Meth daily, or infected with HIV-1 and treated with Meth and IL-1RA (200 or 400 ng/mL) daily. Culture supernatants were analyzed for HIV-1 replication by p24 ELISA, and *p*-values were calculated relative to HIV+ for HIV+Meth samples, or relative to HIV+Meth for HIV+Meth+Il-1RA samples (*p < 0.05, **p < 0.01, ***p < 0.001). **(G)** Cells were harvested at 2 days P.I. IL-1β mRNA expression was analyzed by RT-qPCR. Fold change was calculated by normalizing HIV+Meth or HIV+IL-1RA to HIV+ samples. Data represent the mean ± SD of 3 independent experiments, and *p* values were calculated relative to HIV+ for HIV+Meth samples, and relative to HIV+Meth for HIV+Meth+IL-1RA samples (*p < 0.05, **p < 0.01).

We first confirmed that Meth increases HIV-1 replication in CD4^+^ T-cells, as shown in previous studies (Figure 5A) (5, 54). Next, we assessed the effects of Meth on release of IL-1β under various conditions. Cells were pretreated for 24 h with Meth before exposure to HIV-1; Meth was then administered daily. At 1 day post infection, we observed significantly increased release of IL-1β in cells either exposed to HIV-1 or HIV+Meth (Figure 5B). However, at 2 days post infection, we observed decreased release of IL-1β.

We then analyzed the expression of IL-1β mRNA and miR-146a in Meth, HIV+, and HIV+Meth treated cells. We observed significantly increased IL-1β mRNA levels as well as increased miR-146a expression across all treatments 2 days post infection (Figure 5C).

Next, we assessed the expression of TRAF6 and IRAK1. Interestingly, TRAF6 mRNA levels were unchanged in Meth treated cells, but showed significantly decreased expression in HIV+ and HIV+Meth samples (Figure 5D). In contrast, IRAK1 mRNA showed significantly increased expression in the presence of Meth alone, but significantly decreased expression in HIV+ samples (Figure 5D). When cells were treated with Meth and HIV-1 in combination, IRAK1 mRNA displayed baseline expression (Figure 5D). By Western Blot, we observed decreased TRAF6 protein levels in Meth, HIV+, and HIV+Meth samples (Figure 5E). IRAK1 protein levels showed no change in Meth treated vs. untreated cells, but decreased expression in HIV+ and HIV+Meth treated cells 2 days post infection (Figure 5E).

These data demonstrate that both HIV-1 and Meth increased IL-1β and miR-146a expression. We also observed that HIV-1 inhibited TRAF6 and IRAK1 expression. Unlike Meth treatment, HIV-1 inhibited TRAF6 at the RNA level. Moreover, IRAK1 was inhibited only in HIV-1 infected samples, consistent with previous reports (55). These findings suggest that HIV-1 inhibits expression of TRAF6 and IRAK1 independent of miR-146a expression.

Interestingly, HIV+ and HIV+Meth samples displayed increased extracellular IL-1β levels on day 1 post infection, while IL-1β mRNA levels were significantly increased at day 2 post infection. These data suggest an important role for a Meth mediated IL-1β auto-regulatory feedback loop, which may augment the inflammatory state triggered during HIV-1 infection.

To assess the involvement of Meth induced IL-1β in enhanced HIV-1 replication, we blocked IL-1 signaling using exogenous IL-1RA. Cells were pretreated with IL-1RA and/or Meth, for 24 h before exposure to HIV-1; IL-1RA and Meth were then administered daily. When HIV-1 infected CD4^+^ T-cells were co-treated with IL-1RA and Meth, the effect of Meth on enhancing HIV-1 replication was significantly attenuated in a dose dependent manner. We observed that while 200 ng/mL IL-1RA was sufficient to reduce the effect of Meth on HIV-1 replication, virus replication in these cells was still significantly higher than in HIV+ cells (Figure 5F). However, when cells were treated with higher concentrations of IL-1RA (400 ng/mL) prior to Meth treatment, HIV-1 replication was significantly inhibited (Figure 5F). Notably, when HIV-1 infected CD4^+^ T-cells were treated with IL-1RA alone, there was no change in HIV-1 replication (Figure S1A).

Next, we analyzed IL-1β mRNA expression in HIV-1 infected CD4^+^ T-cells that were untreated, or treated with Meth, or treated with Meth and IL-1RA together. We observed that HIV+Meth+IL-1RA_200ng/mL_ samples showed only slightly increased IL-1β mRNA expression, while IL-1β mRNA expression remained unchanged in HIV+Meth+IL-1RA_400ng/mL_ samples relative to HIV+ controls (Figure 5G). These results are consistent with the pattern we observed during HIV-1 replication, wherein Meth augments IL-1β mRNA and HIV-1 replication, but these effects are slightly reduced in the presence of 200 ng/mL IL-1RA, and completely abrogated in the presence of 400 ng/mL IL-1RA.

Taken together, these results demonstrate that IL-1 signaling is important for Meth mediated effects on HIV-1 replication. Specifically, our results showed that increased IL-1β expression during Meth treatment plays a role in enhanced HIV-1 replication. Further, we observed relatively unchanged expression of miR-146a, TRAF6, and IRAK1 among HIV+, HIV+Meth, HIV+Meth+IL1RA_200ng/mL_, and HIV+Meth+IL-1RA_400ng/mL_ samples (Figures S1B,C), suggesting that an IL-1β positive feedback loop is central to enhanced HIV-1 replication in the presence of Meth.

### IFNα Treatment Inhibits HIV-1 Replication and Disrupts IL-1β and miR-146a Expression

IFNα has been shown to inhibit both HIV-1 replication and IL-1β expression (15, 56). Thus, we next sought to probe the expression of IL-1β and miR-146a expression in IFNα treated HIV-1 infected CD4^+^ T-cells.

Healthy donor CD4^+^ T-cells were infected with HIV-1 alone (HIV+), infected with HIV-1 and treated with Meth (HIV+Meth), or infected with HIV-1 and treated with IFNα (HIV+IFNα). These experimental conditions allowed us to assess changes in IL-1β expression in baseline, enhanced, or diminished HIV-1 infection, respectively.

**Figure 6:**
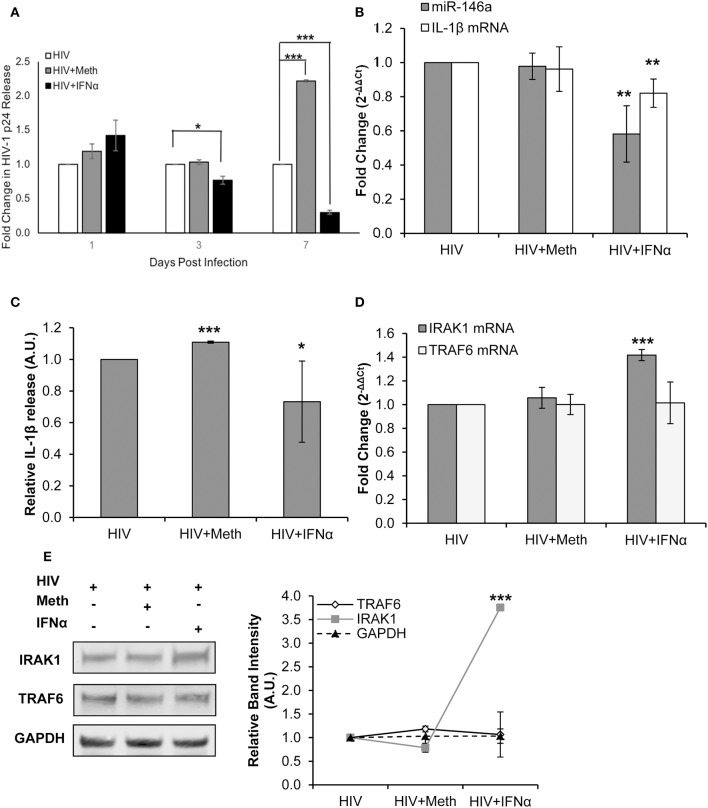
IFNα treatment inhibits HIV-1 replication and disrupts IL-1β and miR-146a expression. CD4^+^ T-cells were infected with HIV-1 alone, infected with HIV-1 and treated with Meth daily, or infected with HIV-1 and treated with IFNα daily. **(A)** Culture supernatants were analyzed for HIV-1 replication by p24 ELISA, and *p*-values were calculated relative to HIV+ controls (*p < 0.05, **p < 0.01, ***p < 0.001). **(B)** Cells were harvested at 2 days P.I. miR-146a and IL-1β mRNA expression was analyzed by RT-qPCR. Fold change was calculated by normalizing HIV+Meth or HIV+IFNα to HIV+ samples. Data represent the mean ± SD of 3 independent experiments, and *p*-values were calculated relative to HIV+ controls (**p < 0.01). **(C)** Culture supernatants were collected 2 days P.I. and analyzed for IL-1β concentration by ELISA. (*p < 0.05, ***p < 0.001). **(D)** Cells harvested at 2 days P.I. were analyzed for TRAF6 and IRAK1 mRNA expression by RT-qPCR. Fold change was calculated by normalizing HIV+Meth or HIV+IFNα to HIV+ samples. Data represent the mean ± SD of 3 independent experiments, and *p*-values were calculated relative to HIV+ controls (***p < 0.001). **(E)** Protein extracts from cells harvested at 2 days P.I. were analyzed for TRAF6 and IRAK1 expression by Western Blot analysis. GAPDH was used as a loading control. Relative band intensity was calculated using ImageJ software, and *p*-values were calculated relative to HIV+ controls (***p < 0.001).

We first confirmed that HIV-1 replication was diminished in the presence of IFNα (Figure 6A). Next, we assessed IL-1β mRNA and miR-146a expression in CD4^+^ T-cells treated under each condition. We observed, relative to HIV+ cells, significantly decreased miR-146a and IL-1β mRNA expression with IFNα treatment (Figure 6B). There was significantly increased IL-1β release in HIV+Meth treated cells, and significantly decreased IL-1β release in HIV+IFNα cells relative to HIV+ cells (Figure 6C). TRAF6 and IRAK1 mRNA expression were unchanged in HIV+ and HIV+Meth conditions, but significantly increased expression of IRAK1 mRNA was observed in cells treated with HIV+IFNα (Figure 6D). Finally, TRAF6 protein was maintained at low levels of expression among HIV+, HIV+Meth and HIV+IFNα samples (Figure 6E). However, IRAK1 protein showed increased expression in cells treated with HIV+IFNα compared to both HIV+ and HIV+Meth treated cells (Figure 6E).

Taken together, these results demonstrate that immunomodulation by IFNα inhibited HIV-1 replication concordantly with decreased IL-1β and miR-146a expression in CD4^+^ T-cells. Interestingly, TRAF6 protein and mRNA levels were unchanged among HIV+, HIV+Meth, and HIV+IFNα samples. Moreover, IFNα significantly increased the expression of IRAK1 at RNA and protein levels concordant with decreased miR-146a expression.

## Discussion

Meth abuse is a well-established risk factor for HIV/AIDS (5, 57). The drug can enhance virus replication and clinical progression, often promoting poor adherence to anti-retroviral therapies. Meth has also been shown to modulate inflammatory cytokine expression (10–12). Specifically, Meth has been reported to enhance IL-1β expression in dendritic cells and the rat hypothalamus (13, 14). The effects of Meth on host defenses and the miRNAs that regulate these processes, are not well-defined (3–5, 9, 18, 23, 58). In addition, miRNA targets are highly sensitive to changes in their expression (23, 24). IL-1β has been shown to participate in an auto-regulatory loop, and stimulate NFκB-dependent miR-146a to disrupt key inflammatory responses (31, 43). Several direct targets of miR-146a have been implicated in HIV-1 pathobiology (47, 48, 59). Notably, miR-146a targets both TRAF6 and IRAK1, signaling molecules that facilitate innate immune responses (26, 27, 48, 60). HIV-1 can induce expression of IL-1β, which is associated with progression of HIV/AIDS, and in microglial cells HIV-1 infection resulted in overexpression of miR-146a (16, 61).

Here, we demonstrate that Meth can induce expression of IL-1β and miR-146a in CD4^+^ T-cells, with overexpression of the cytokine leading to increased expression of miR-146a. Meth mediated miR-146a overexpression targeted TRAF6 to modulate innate immune signaling pathways. Based on our findings, we hypothesized that IL-1β signaling results in increased IL-1β mRNA levels and increased miR-146a expression. We further hypothesized that IL-1β-dependent induction of miR-146a would target key innate immune pathways by decreasing TRAF6 expression (Figure S2). Although there are several known targets of miR-146a, only TRAF6 was significantly affected during Meth treatment. We also observed that induction of an IL-1β auto-regulatory loop contributed to Meth mediated increases in HIV-1 replication.

Meth treatment of CD4^+^ T-cells augmented Caspase-1 activation and enhanced IL-1β release. Subsequently, both IL-1β mRNA and miR-146a levels rose, indicating that IL-1β increased expression of these transcripts. We also explored changes in TRAF6 and IRAK1 proteins, both direct targets of miR-146a. While we found no change in IRAK1 protein expression, there was decreased TRAF6 expression, indicating selective inhibition of TRAF6 by Meth via miR-146a.

To further elucidate the role of Meth in IL-1β and miR-146a overexpression, we blocked IL-1β signaling at two steps. Cells were treated with either IL-1RA to block binding of IL-1β to its receptor, or exogenous IFNα, a type I IFN known to inhibit Caspase-1 activation and antagonize mature IL-1β release. We observed decreased IL-1β release after exogenous IFNα treatment, along with decreased Caspase-1 activation. Blocking either the IL-1 receptor with IL-1RA, or release of IL-1β with IFNα, abrogated Meth mediated effects on miR-146a and IL-1β mRNA. Our data demonstrate that Caspase-1 activation, and increased IL-1β release and signaling are critical for Meth mediated miR-146a overexpression and enhanced IL-1β mRNA expression. Notably, Caspase-1 and IL-1β are associated with pryoptosis, or inflammation mediated apoptosis; pryoptosis is linked to progression of HIV-1 infection via CD4^+^ T-cell depletion (62–64).

At day 1 of HIV-1 infection, we observed enhanced release of IL-1β, followed by increased IL-1β mRNA and miR-146a expression at day two. Decreased TRAF6 expression, a known miR-146a target, is associated with increased HIV-1 replication (47). We observed decreased TRAF6 expression upon infection with HIV-1 at the gene expression level. IRAK1 protein expression levels were also decreased following HIV-1 infection. These findings are consistent with previous reports that HIV-1 decreases IRAK1 and TRAF6 expression (47, 55).

In sum, we found that Meth enhanced HIV-1 replication through an IL-1β positive auto-regulatory loop. By blocking IL-1 signaling using IL-1RA, we observed dose dependent decreased HIV-1 replication after Meth treatment. We also observed that Meth enhanced IL-1β mRNA levels were abrogated in a dose dependent manner upon treatment with IL-1RA. These results support the involvement of an IL-1β auto-regulatory loop in Meth mediated enhanced HIV-1 replication. Alternatively, when HIV-1 replication was inhibited using IFNα, we found decreased IL-1β and miR-146a expression. Our results indicate that increased levels of IL-1β directly contribute to Meth mediated increased HIV-1 replication in CD4^+^ T-cells. Based on our results, it appears that Meth mediated increased IL-1β expression acts to prime cells to be more susceptible to infection with HIV-1.

These studies unveil unique effects of Meth on IL-1β to dysregulate innate immune signaling pathways and enhance HIV-1 infection. This novel mechanism of action of Meth points to potential therapeutic targets relevant to drug abusing hosts.

## Data Availability Statement

All datasets generated for this study are included in the article/Supplementary Material.

## Ethics Statement

The studies involving human participants were reviewed and approved by Beth Israel Deaconess Medical Center Committee on Clinical Investigations (CCI). Written informed consent for participation was not required for this study in accordance with the national legislation and the institutional requirements.

## Author Contributions

KL: conceptualization, investigation, and writing. AP: methodology and writing. JG: supervision, funding acquisition, and writing.

### Conflict of Interest

The authors declare that the research was conducted in the absence of any commercial or financial relationships that could be construed as a potential conflict of interest.

## Supplementary Material

Figure S1 No effect of IL-1RA on HIV-1 replication, TRAF6, miR-146a, or IRAK1 expression. CD4^+^ T-cells were infected with HIV-1 alone, or infected with HIV-1 and treated with Meth or IL-1RA at varying concentrations. **(A)** Culture supernatants were analyzed for HIV-1 replication by p24 ELISA. Statistical analyses were performed to assess changes relative to HIV+ controls. No significant changes were observed. **(B)** miR-146a expression was analyzed by RT-qPCR. Fold change was calculated by normalizing HIV+Meth, HIV+IL-1RA200 ng/mL, and HIV+IL-1RA400 ng/mL to HIV+ samples. Data represent the mean ± SD of 3 independent experiments (^***^*p* < 0.001). *p*-values were calculated relative to HIV+ controls. **(C)** Protein extracts from cells harvested at 2 days P.I. were analyzed for TRAF6 and IRAK1 expression by Western Blot analysis. GAPDH was used as a loading control. Relative band intensity was calculated using ImageJ software (^***^*p* < 0.001). *p*-values were calculated relative to untreated controls.

Figure S2 Hypothetical relationship between IL-1β signaling and miR-146a expression and inhibition of TRAF6. Visual representation of hypothesized pathway for Meth induced effects on miR-146a and downstream targets via IL-1β.
