# Supplementary material for: Methamphetamine Enhances HIV-1 Replication in CD4+ T-Cells via a Novel IL-1β Auto-Regulatory Loop
Source: Front Immunol. 2020 Feb 7;11:136. doi: 10.3389/fimmu.2020.00136 (PMC7025468; doi:10.3389/fimmu.2020.00136)

Supplementary Figure S1-No effect of IL-1RA on HIV replication, TRAF6, miR-146a, or IRAK1 expression.

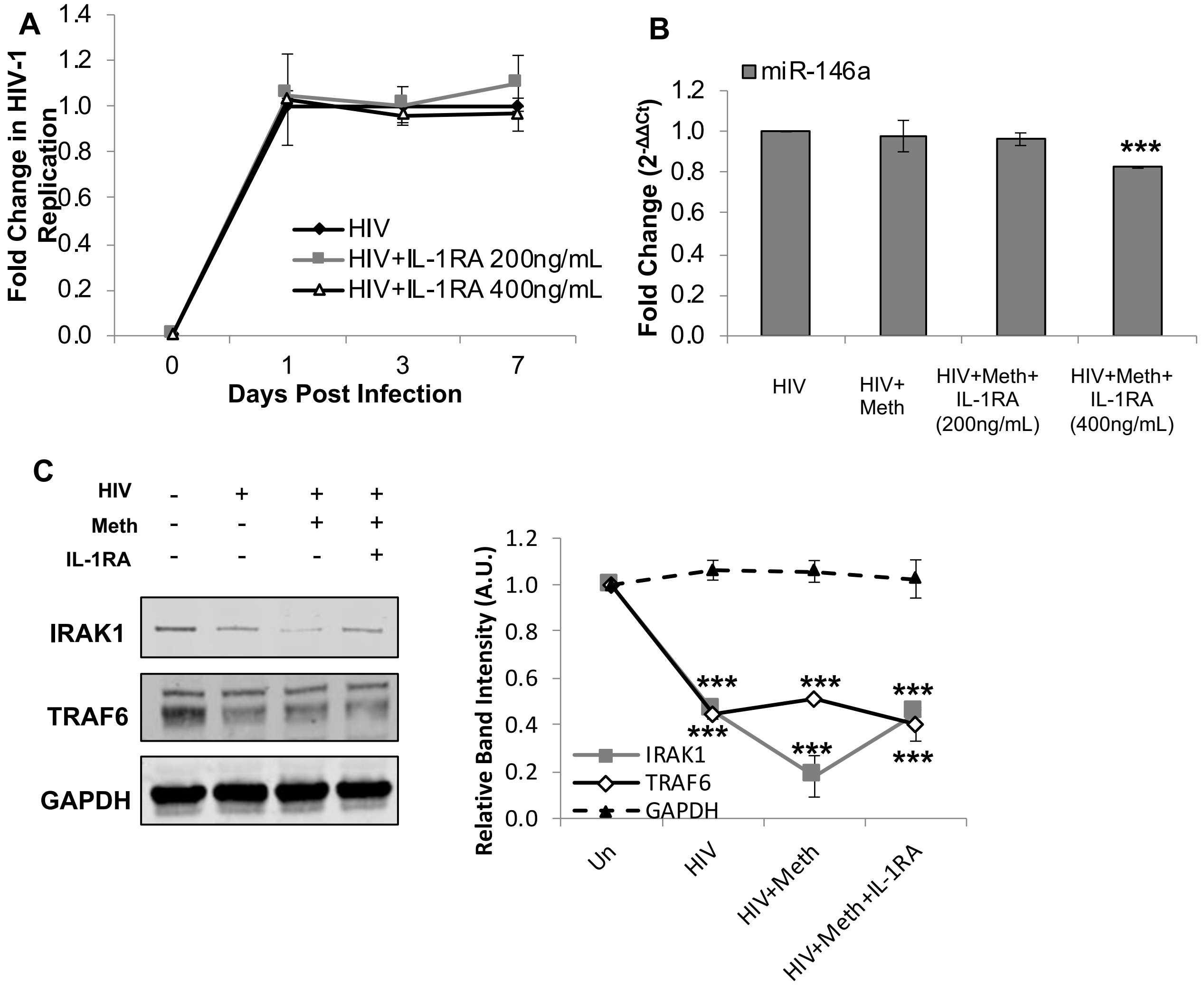

Supplementary Figure S2- Hypothetical relationship between IL-1ß signaling and miR-146a expression and inhibition of TRAF6.

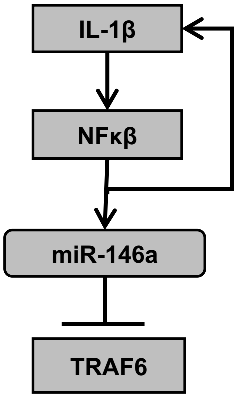

Supplement: Figure S1 — No effect of IL-1RA on HIV-1 replication, TRAF6, miR-146a, or IRAK1 expression. CD4+ T-cells were infected with HIV-1 alone, or infected with HIV-1 and treated with Meth or IL-1RA at varying concentrations. (A) Culture supernatants were analyzed for HIV-1 replication by p24 ELISA. Statistical analyses were performed to assess changes relative to HIV+ controls. No significant changes were observed. (B) miR-146a expression was analyzed by RT-qPCR. Fold change was calculated by normalizing HIV+Meth, HIV+IL-1RA200 ng/mL, and HIV+IL-1RA400 ng/mL to HIV+ samples. Data represent the mean ± SD of 3 independent experiments (***p < 0.001). p-values were calculated relative to HIV+ controls. (C) Protein extracts from cells harvested at 2 days P.I. were analyzed for TRAF6 and IRAK1 expression by Western Blot analysis. GAPDH was used as a loading control. Relative band intensity was calculated using ImageJ software (***p < 0.001). p-values were calculated relative to untreated controls. [file Data_Sheet_1.PDF]
